# Perception and application of pre-operative surgical antibiotic prophylaxis by physicians

**DOI:** 10.6026/973206300210647

**Published:** 2025-04-30

**Authors:** Mohammed Jaffer Ali, Madeeha Hussaini, Omkumar M. Patel, Mohammad Faisal Uddin, Mohamed Ali, Asad Alnahar, Lulyah Almallah, Yasir Adil El Rashid Mohamed, Hamza A. Orfali, Mohammed Abdul Mateen

**Affiliations:** 1 Department of Internal Medicine, Bhaskar Medical College, Hyderabad, India; 2 Department of Internal Medicine, Shadan Institute of Medical Sciences, Hyderabad, India; 3 Department of Internal Medicine, University of Northern Philippines, Philippines; 4 Department of Acute Medicine and medicine of the elderly, Victoria Hospital, Kirkcaldy, United Kingdom; 5 Department of General Medicine, Atlas International Medical Complex, Riyadh, Saudi Arabia; 6 Medical Intern, Vision College, Riyadh, Saudi Arabia; 7 Department of Medicine, Military Medical Hospital, Omdurman, Khartoum, Sudan; 8 Department of Medicine, Al Neelain University, Sudan

**Keywords:** Surgical site infections (SSIs), surgical antimicrobial prophylaxis, physicians

## Abstract

Surgical site infections (SSIs) are the most common and prevalent complications occurring post-operatively leading to additional costs to the health care system. Hence, medical interns, general practitioners, surgical residents and surgeons who meet the inclusion criteria were included in this cross-sectional study. Surgical antimicrobial prophylaxis (SAP) was recorded using a structured questionnaire. Data shows that senior surgeons, scored highest and of the 21 surgical residents, 25 medical interns and 6 GPs the performances varied with overall averages of 84.37%, 76.56% and 34.37%, respectively. This implies that medical practitioners demonstrate robust medical knowledge and practical skills, but there is scope to cultivate their professional conduct and interpersonal competencies.

## Background:

Surgical site infection (SSI) is a term described as an infection that arises post 30 days after the surgery, or within a year in patients with implants, affecting incision site and deeper tissues [1]. Despite various advancements, surgical site infections (SSIs) is a significant clinical challenge due to its association with high mortality and morbidity rates, as well as their substantial impact on healthcare resources [2]. SSIs are known to increase patient complications, resulting in longer hospital stays and even death which causes serious impact on patient outcomes. Treating SSIs requires longer hospital admissions, additional medical interventions and increased use of antibiotics which place a significant burden on healthcare system. Healthcare provider including patients, experience significant economic effects due to the high cost of treating SSIs, which may include prolonged hospital stays and additional procedures, for management of complications. The occurrence of SSIs can vary till 20%, depending on factors such as the surgical procedure, repetitiveness, follow-up criteria and the quality of data collection [3]. The specific pathogens causing surgical site infections (SSIs) are typically dependent on the type of surgery performed on the patient. However, the most common causative agents are generally Staphylococci, Enterococcus species and Escherichia coli [4]. These pathogens are commonly found in the skin, gastrointestinal tract and other areas and can easily contaminate surgical sites, leading to the development of SSIs. The prevalence of these specific microorganisms in causing SSIs highlights the importance of proper surgical techniques, infection control measures and appropriate antibiotic stewardship to prevent and manage these serious healthcare-associated infections [5]. Both patient-related and procedure-related factors contribute to the risk of SSIs; hence, there is a need for a bundled strategy for prevention that targets all possible risk factors by achieving a reduction in bacterial load and enhancing the patient's immunity [6]. New technologies, which include microbial sealants, help in preventing or containing skin organisms during operation, further helping the argument for assessing these technologies and maybe even using them in regular practice [7]. Therefore, it is of interest to discuss the perception and application of pre-operative surgical antibiotic prophylaxis by physicians.

## Materials and Methodology:

## Study design:

A cross-sectional study was held from January 19 to June 23, 2023, at the General Surgery unit of a tertiary teaching hospital, during which antimicrobial prophylaxis was monitored in 223 elective general surgery lines of action. Data collection for antibiotic prophylactic therapy was directly withdrawn from the patient's status.

## Sample size:

The sample size was calculated with KAP analysis concerning a previous study conducted by in 2015 [8]. The selection of participants was done that included various departments within the field of surgery, including cardiology, gastroenterology and pulmonology. The sample size of 64 was determined using the power of 80% and a margin of error of 5%.

## Declaration of ethics:

The study received ethical clearance of tertiary teaching hospital Institutional Review Board under protocol number TCTH/204/309 and ethical clearance was received. Written informed consent was required from doctors and the Data confidentiality was protected. The respondents' choice to decline an interview was honored.

## Source population:

The study population comprises of all available and willing members of the medical staff at the healthcare facility over the period data was collected. The inclusion criteria for the study were that any doctor who agreed to participate was eligible for enrolment such as a medical intern, as they carried out antimicrobial prophylaxis on the surgical components, General practitioners (GPs) in surgery wards, surgical residents and surgeons were also asked to complete the self-administered questionnaire. Conversely, those who were unwilling to take part were excluded. Ultimately, 64 participants were included in the final study population. This sample was selected to provide a representative cross-section of the healthcare providers at the facility, minimizing potential bias. Before data collection, the Institutional Review Board (IRB) reviewed and approved the study protocol and methods. All participants provided informed consent before taking part in the research. These measures were taken to ensure the ethical conduct and protect the rights and well-being of the healthcare providers involved.

## Procedure:

A cross-sectional survey was conducted to assess surgeons' awareness and compliance with evidence-based guidelines for antimicrobial prophylaxis by a cross-sectional survey. The survey questionnaires are designed depending on the guidelines from the national institute for health and care excellence (NICE), world health organization (WHO) standard, Stanford Health Care (SHC). The questionnaire had two sections; the first section included demographic data such as age, sex, education level and medical specialty. The second part consists of assessing the awareness about surgical procedures in which Standardised Antibiotic Prophylaxis (SAP) is indicated, type of antibiotics for different surgical procedures and the sources through which they obtain information regarding SAP. The third section evaluated the participants' attitudes towards the development of national SAP guidelines and their willingness to support such initiatives. The final section examined the extent of physician compliance with SAP adherence. The questionnaire was distributed to a convenient sample of senior surgeons, surgical residents, medical interns and general practitioners. The collected data was analysed to determine the surgeons' knowledge, attitudes and adherence to antimicrobial prophylaxis guidelines.

## Data interpretation:

According to the 2013 SAP guideline from the American Society of Health System Pharmacists (ASHP), all responses received a score of 0 for all other answers, while a score of 1 was given for all correctly answered knowledge and practice questions. The answers were "never" for negative questions and "always" for positive questions. In relation to the guidelines, 5 specify strongly agreement, 4 agreements, 3 neutrality, 2 disagreements and 1 for strongly disagreement for positive questions and vice versa for the negative ones. A higher than the average score of the mean is indicative of a good attitude. However, for the remaining attitude questions, if the response complies with the evidence-based surgical antimicrobial prophylactic advice, one point is awarded; if not, zero points are awarded.

## Results:

Ninety-nine questionnaires were delivered in the first phase; 62 were returned to the data collectors, with three completely blank, two lacking the attitude and practice sections and one lacking the sociodemographic component. Five questionnaires completed by clinicians who did not participate in the initial day of data collection were collected on different days during the ward rotation (Table 1). The study involved 64 participants out of which the majority were male 87.5% (n=56) and the remaining 12.5% (n=8) were female. The age of the participants ranged from 23 to 40 years, with the majority (48.42%, n=31) being between 26 to 30 years old, followed by 42.18% (n=27) in the 23 to 25 age group and 9.4% (n=6) in the 31 to 40 age group. Regarding the organizations the participants were working for, the majority (81.25%, n=52) tertiary teaching hospital, University Medical Center, while the remaining 18.75% (n=12) worked at associated settings. In terms of experience, 57.81% (n=37) of the participants had ≤2 years of experience, 23.44% (n=15) had 3-5 years of experience and 18.75% (n=12) had ≥6 years of experience. The practice level of the participants was diverse, with 32.81% (n=21) being surgery residents, 39.06% (n=25) being medical interns in surgical rotation, 9.38% (n=6) being general practitioners and 18.75% (n=12) being senior surgeons. Regarding the frequency of prescribing antibiotic orders in the last week, 35.94% (n=23) of the participants reported 'always' prescribing antibiotics, 32.82% (n=21) reported 'often', 25% (n=16) reported 'sometimes', 3.12% (n=2) reported 'seldom' and 3.12% (n=2) reported 'never' prescribing antibiotics.

The overall correct answer rate for knowledge-related items was 84.37%. Senior surgeons had the highest correct answer rate at 100% for two of the knowledge items (Q1 and Q3). Surgical residents also performed well, with correct answer rates ranging from 93.33% to 20% across the knowledge items. Medical interns had the lowest correct answer rates, ranging from 68% to 8% across the knowledge items. General practitioners had correct answer rates between 100% and 21.87% for the knowledge items as mentioned in (Table 2). It was determined that practice related items have a correct response rate of 76.56%. General practitioners had the highest correct answer rate at 100% for one of the practice items (Q4). Surgical residents and medical interns also performed well, with correct answer rates ranging from 100% to 32% across the practice items. Senior surgeons had corrected answer rates between 100% and 33.33% for the practice items as mentioned in Table 2. The overall correct answer rate for attitude-related items was 28.13%. General practitioners, senior surgeons and medical interns all had the highest correct answer rates, ranging from 40% to 16.67% across the attitude items. Surgical residents had the lowest correct answer rates, ranging from 32% to 16.67% for the attitude items as mentioned in Table 2.

## Discussion:

Surgical site infections (SSIs) are a prevalent and severe consequence of surgical operations, resulting in higher rates of illness, death and healthcare services expenses [6]. Surgical antibiotic prophylaxis (SAP) is a proven and scientifically supported method to avoid surgical site infections (SSIs) [7]. Nevertheless, the existing data indicates that there are substantial disparities between the intended recommendations for surgical procedures and the real understanding and behaviours of surgeons in different healthcare environments. The current study challenges multiple prior studies and emphasizes that surgeons typically have a favourable view of the necessity of local and national guidelines on surgical anaesthesia management (SAP) [8]. Furthermore, their understanding and compliance with these recommendations are considered to be adequate. The findings indicated that medical professionals generally possess a robust understanding of the necessary information for their professions. Among them, senior surgeons and surgical residents exhibited the highest degrees of competence based on knowledge analysis. This is indicative of their comprehensive training and expertise in the domain. This study, however, refutes the previous claims [9], which showed that very few (12.5%) of the surveyed surgeons had a deep comprehension of the advisable surgical anaesthesia processes and procedures (SAP). The acquired knowledge suggests that there are a good number of surgeons who have the requisite knowledge and therefore would be able to make rational decisions on surgical procedures, which could eliminate the chances of surgical site infection (SSIs). Most of the previous studies both focused on the knowledge aspects and lack of any knowledge and the lack of practical ways to implement, in their clinical work, the recommendations regarding the SAP [10].

As far as practice-related issues are concerned, it was observed that the performance was quite good even among practitioners of all levels, with general practitioners, surgical residents and medical interns scoring the highest. Hence, these practitioners are equipped with the knowledge and skills to put their knowledge to good use in a clinical setting. Previous studies indicated that such practices are not always performed according to the recommended best practices [11]. Relatively common errors include the incorrect timing of the antibiotic doses, deviations from the rules for the intraoperative dose and the use of inappropriate antibiotics. Another study sought to find out the level of understanding of SAP among thoracic surgeons and found out that 70% understood the concept of SAP yet less than half understood the proper antiseptic precautions to take for patients prone to gram-negative bacteria and penicillin allergy [12]. At the same time, it should be emphasized that only 40% of lung surgeons have adequately followed the prescribed antibiotic regimens, underscoring the further improvement of compliance with guideline recommendations.

Surgeons might consider it acceptable to focus on more channels of the surgical process than strictly observing the prescribed procedural standards of SAP. Likewise, there are studies which dispassionately proved that discomfort or reluctance on the side of the surgeon affects the appropriate and timely definitive management of SAP [13]. Some surgeons could refrain from fully complying with such recommendations, opting for a more conservative approach than warranted such as sweeping antibiotics or prolonged prophylactic treatment. In doing so the intention is more of protecting their patients; however, such conduct could in future lead to inappropriate antibiotic uses and thus antimicrobial resistance development. It has also been noted that some of the differences in the practice are also as a result of lack of specific national and local policy on SAP [14]. Nonetheless, the lower percentages of correct responses observed in regard to the items that relate to attitudes specifically suggest that there is room for improvement in the cultivation of soft skills and professional attitudes of medical practitioners [15]. This is an area where both healthcare organizations and education institutions need to focus on to ensure that, in addition to the requisite knowledge and skills, practitioners are able to exhibit appropriate professional conduct and attitudes that are indispensable in provision of quality care to patients. The gap between the surgical knowledge and what practitioners actually do in clinical practice underscores an even more complex set of barriers that calls for a broader, multi-faceted intervention strategy. This may require more focused action to change cultures, train and support healthcare staff, as well as align any incentives and policies which need to be addressed in order for evidence-based practices1 (EBPs)2 to flow more easily into ushering EBPs into standard clinical practice. Removing these barriers is crucial for the development of patient management and also for alleviating the effects of SSIs [16]. The approach to filling the gaps that exist in both problem formulation about the identification of SAP conflicts among surgeons and the identification of structure process such as a lack of norms surrounding surgical anatomical positioning (SAP) at local or national levels may include development, dissemination of evidence-based guidelines [17] and attitude and hierarchy issues [9] and some recommendations on specific roles for various working groups in counteracting these errors have been elaborated. They should be developed with input from different groups of staff members, but not limited to infectious disease specialists, pharmacists and surgeons [18]. This method will guarantee that the guidelines will be feasible, easy-to-use and applicable to particular concerns of local health care system [19]. This approach will ensure that the guidelines will be practical, straightforward and relevant to the specific needs and challenges of the local health care system. Once developed the recommendations should be made widely available and promoted within surgical practice. This may involve awareness campaigns, training activities and inclusion of SAP values in the policies and procedures of the Organization.

## Strength and limitations:

In addition, the survey was itself developed based on established international guidelines from various organizations, thus ensuring that questions were grounded in best practices. Additionally, the diverse inclusion of participants from different surgical roles, including medical interns, surgical residents, general practitioners and senior surgeons, offers a broad perspective on various levels of knowledge and adherence across the surgical team. Given that this study is also a teaching hospital, it is able to represent the clinical phenomenon and high volume of surgery in this context. As strength, the design of this study deserves note. Cross-sectional studies, such as that found in this report, have a major benefit of being able to collect many data points at one time. Conducted in a teaching hospital, the study reflects real-world clinical practices in a high-volume surgical environment, enhancing its relevance to similar healthcare settings. It is a further strength of this study that it was includes a large number of different type's decision-makers. Furthermore, the study adhered to strict ethical standards, with Institutional Review Board (IRB) approval and informed consent from participants, ensuring that the rights and confidentiality of healthcare providers were protected.

However, the research also has drawbacks that affect its generalisability. Convenience sampling could introduce selection bias because the sample body might not be entirely representative of the wider population of healthcare providers, in turn skewing results toward those who are more readily available or willing to join a research project. In addition, the relatively small sample size of 64 did not permit generalization of the results, as a larger number of subjects would provide more credible information. Furthermore, the study may be affected by response bias. This is particularly the case if the questionnaires are completed without supervision, as participants can then consult external resources and distort their recorded knowledge to get rid of any inaccuracies. Moreover, social desirability bias might have influenced participants to overstate their adherence to antimicrobial guidelines. Another limitation is the lack of specificity in some survey questions. Some questions in the questionnaire could not offer specific data. For instance "how often do you take antibiotics?" "Sometimes" and are of no help at all. This is a problem concerning the validity of findings which needs to be considered when the results are applied beyond their immediate limits.

## Conclusions:

The skills associated with the knowledge and practice of medical practitioners, as well as their attitudes is reported. Data shows that medical practitioners in general have enough knowledge and skills regarding the employment places. Nevertheless, improvement of their work ethics and soft skills can be further improved. The impressive performance of experienced surgeons and surgical residents in knowledge-based tests demonstrates the efficacy of the rigorous training and educational programs such professionals undergo.

## Figures and Tables

**Table 1:** Socio socio-demographic characteristic of respondent

**Variable**	**Categories**	**Frequency**	**%**
Gender	Male	56	87.5
	Female	8	12.5
Age	23 to 25	27	42.18
	26 to 30	31	48.42
	31 to 40	6	9.4
Organization	Tertiary care center	52	81.25
	Associated centers	12	18.75
Experience in years	≤ 2	37	57.81
	5-Mar	15	23.44
	≥ 6	12	18.75
Practice Domain	Senior Surgeon	12	18.75
	Surgery Resident	21	32.81
	Medical intern	25	39.06
	General Practitioner	6	9.38
Prescription order > 1 week	Always	23	35.94
	Often	21	32.82
	Sometimes	16	25
	Seldom	2	3.12
	Never	2	3.12

**Table 2:** Score for knowledge, practice and attitude questions

	**Answers given by the level of medical Practitioner**				N=64 Total n (%)
	**Position and Practice status**				
	**N=3 Senior Surgeon n (%)**	**N=30 Surgical Resident n (%)**	**N=25 Medical Intern (%)**	**N=6 General practitioners (%)**	
**Knowledge related items**					
Question	3(100)	28 (93.33)	17(68)	6(100)	54(84.37)
Question 2	2(66.67)	22(73.33)	14(56)	5(83.33)	43(67.19)
Question 3	3(100)	29(96.67)	19(76)	6(100)	57(89.06)
Question 4	2(66.67)	6(20)	2(8)	4(66.67)	14(21.87)
Question 5	2(66.67)	10(33.33)	5(20)	4(66.67)	21(32.81)
Question 6	1(33.33)	23(76.67	13(52)	3(50)	40(62.5)
**Practice related items**					
Question 1	2(66.67)	24(80)	19(76)	6(100)	49(76.56)
Question 2	2(66.67)	18(60)	16(64)	5(83.33)	41(64.06)
Question 3	3(100)	21(70)	21(84)	5(83.33)	50(78.12)
Question 4	2(66.67)	27(90)	25(100)	6(100)	60(93.75)
Question 5	1(33.33)	10(33.33)	9(36)	3(50)	13(20.31)
Question 6	3(100)	17(56.67)	8(32)	4(66,67)	32(50)
Question 7	1(33.33)	11(36.67)	6(24)	5(83.33)	23(35.93)
**Attitude related items**					
Question 1	1(33.33)	8(26.67)	5(20)	2(33.33)	16(25)
Question 2	2(66.67)	5(16.67)	7(28)	1(16.67)	15(23.44)
Question 3	1(33.33)	8(26.67)	8(32)	2(33.33)	19(29.69)
Question 4	1(33.33)	9(30)	10(40)	2(33.33)	22(34.37)
